# Promotion of Wellbeing for Children of Parents With Mental Illness: A Model Protocol for Research and Intervention

**DOI:** 10.3389/fpsyt.2019.00606

**Published:** 2019-09-06

**Authors:** Charlotte Reedtz, Karin van Doesum, Giulia Signorini, Camilla Lauritzen, Therese van Amelsvoort, Floor van Santvoort, Allan H. Young, Philippe Conus, Richard Musil, Thomas Schulze, Michael Berk, Argyris Stringaris, Geneviève Piché, Giovanni de Girolamo

**Affiliations:** ^1^RKBU North, Faculty of Health, UiT – Arctic University of Norway, Tromsø, Norway; ^2^Team Preventie, Onderzoeker Mindfit, Nijmegen, Netherlands; ^3^Unit of Epidemiological and Evaluation Psychiatry, IRCCS Istituto Centro San Giovanni di Dio Fatebenefratelli, Brescia, Italy; ^4^Department of Psychiatry and Psychology, Maastricht University, Maastricht, Netherlands; ^5^Pluryn/Hoenderloogroep, Research & Development, Nijmegen, Nederlands; ^6^Department of Psychological Medicine, Institute of Psychiatry, Psychology and Neuroscience, King’s College London and South London and Maudsley NHS Foundation Trust, London, United Kingdom; ^7^Service de Psychiatrie Générale, Dép. de Psychiatrie, Centre Hospitalier Universitaire Vaudois (CHUV), Lausanne, Switzerland; ^8^Bipolar Disorders and Borderline Personality Disorders Unit, Clinic for Psychiatry and Psychotherapy, LMU Klinikum der Universität München, München, Germany; ^9^Institute for Psychiatric Phenomics and Genomics (IPPG), Klinikum der Universität München, München, Germany; ^10^IMPACT Strategic Research Centre, School of Medicine, Barwon Health, Deakin University, Melbourne, VIC, Australia; ^11^Orygen Youth Health Research Centre and the Centre of Youth Mental Health, The Florey Institute for Neuroscience and Mental Health and the Department of Psychiatry, University of Melbourne, Melbourne, VIC, Australia; ^12^Mood and Development Laboratory, Emotion and Development Branch, National Institute of Mental Health, National Institutes of Health, Bethesda, MD, United States; ^13^Psychoeducation and Psychology Department, Université du Québec en Outaouais, Gatineau, QC, Canada

**Keywords:** children of parents with mental disorders, mental health care for adults, risk and protective factors, wellbeing, prevention

## Abstract

**Background:** The main objective of this project is to create a research and intervention model to promote large-scale implementation and evaluations of generic very brief interventions for children of parents with mental disorders (COPMI). Feasible interventions for COPMI aged 0–18 years are highly needed, as this is a large high-risk group in society. Reducing behavioral problems and enhancing wellbeing for families with parents affected by any mental disorder are important preventive initiatives. One key prevention strategy is to reduce the risk and expression of psychopathology in children and to promote wellbeing. The present model protocol offers an intervention for children of parents with mental disorders internationally based on a model already implemented in the Netherlands and Norway.

**Methods:** Participants will be parents receiving treatment in mental health services in participating countries and their minor children aged 6–18 years. Participants should be randomized into an intervention group or control group. Data should be retrieved from electronic patient journals (demographics, DSM 5/ICD-10, SCID, MINI) as well as from assessment measures administered at baseline and follow-up, including the KIDSCREEN-27, Strengths and Difficulties Questionnaire (SDQ), Parents’ Evaluations of Developmental Status (PEDS), Parenting Sense of Competence (PSOC), Resilience Scale for Adolescence (READ), Guilt and Shame Questionnaire for Adolescents of Parents with Mental Illness (GSQ-APMI), Mental Health Literacy Scale, and Parent–Child Communication Scale.

**Results:** The hypothesis is that there will be improvements of child behavioral and emotional problems, and outcomes in the project will be reported in terms of parent´s diagnosis, child behavioral and emotional problems, child wellbeing, family communication and functioning, as well as participants’ satisfaction.

**Discussion:** This multi-site international protocol will focus the attention of European scientific and policy makers toward COPMI. This young segment of the population is presently almost completely neglected in most European health policies, despite having a large burden of disability and being at risk of transgenerational transmission of psychopathology. We will further discuss the feasibility of a very brief intervention aiming at preventing mental disorders in young people.

## Introduction

Mental disorders in parents are a major biological and environmental risk factor to which many young people are exposed. About 15–23% of children live with a parent with a mental disorder worldwide (1, 2). These children are 5.2 times more at risk of depression and 3.7 times more at risk of anxiety disorders compared to their peers (3). Children of parents with mental disorders are also at risk of poorer intellectual and social outcomes (4), affect dysregulation (5), behavioral problems (6), impaired attention, and reduced overall adaptive functioning (7). Research has also shown that children of parents with mental disorders have higher rates of substance abuse and multiple diagnoses (8), as well as a lower occupational status. The transmission of parental psychopathology to children can lead to similar (transgenerational equi-finality) as well as different (transgenerational multi-finality) clinical outcomes than their parent’s diagnosis (9–11). The risk factors and adverse outcomes create an amplifying vicious cycle, mandating preventative action (12).

Living with a parent suffering from a mental disorder may imply the exposure to a variety of risk conditions, including: a) an adverse family environment characterized by poor parenting, high stress reactivity, emotional vulnerability, and compromised family functioning; b) experience of guilt, stigma, shame and loneliness, and perceptions of lacking social support and social acceptance; and c) the reversal of care-giving (“parentification”) (13). According to Hosman and colleagues (14), the adverse outcomes for these children are the result of a complex interplay between three systemic levels (parent, child, and parent–child relationship) and potential risk factors (such as parents’ mental disorder, lack of social support, poor financial conditions, marital discord, etc.). Research has shown that impaired parent–child interaction is the environmental risk factor that explains most variance in child psychological problems (27.2%) (15).

However, through carefully designed interventions addressing this interplay, one can enhance wellbeing for families with parents suffering from a mental disorder. Interventions aiming at reducing the impact of risk factors within the family context and strengthening children’s ability to cope are promising, although the results in terms of effect size seem still of limited amplitude. A meta-analysis, from 2012 on 13 trials on preventive interventions for mental disorders in offspring, indicated how the risk of developing parents’ mental disorder could be decreased by 40% (16). In a recent meta-analysis, 50 randomized controlled trials on preventive interventions for mental disorders in offspring showed small, but significant effect sizes, stable over 12-month follow-up, for programs enhancing the mother–child interaction (17). Interventions addressing parents and children jointly produced overall larger effects. Nevertheless, the conclusion of this review was that there is a scarcity of high-quality studies that effectively reduce the high risk of COPMI for the development of mental disorders.

Targeting children at risk and approaching them through their parents, rather than waiting until they become adults, will strengthen the reach of youngsters at their most critical point for the onset of mental disorders (18). Some research has explored experiences of youngsters living in these families and their expressed needs (19), and results show that minor children want more information about their parent’s disorder and practical support related to coping with the family situation. Moreover, children of parents with a mental disorder should be expected to be as heterogeneous as the group of their parents. In order to understand their life lived experiences and needs, health professionals and social workers therefore need to listen to them. Preventive intervention could provide more complete and effective care provision for parents already in care (20). Including a whole family approach could increase treatment outcomes in adult mental health care as well as patient satisfaction with service providers. For these reasons, healthcare systems seriously need to consider preventive programs for children of mentally ill parents.

As suggested by a pioneering Australian example, it is imperative that such programs are offered to the public once their effectiveness has been established in terms of immediate and long-term outcomes (21), through both quantitative and qualitative data (22). Interventions should be designed to reduce the impact from stress and lack of quality care within the family context (related to poor or absent communication, poor understanding of disorders, insufficient parental care, lack of clinical support, or negative attitudes toward help-seeking, etc.), as well as to strengthening children’s ability to cope by informing them of their parents’ mental disorder and to supply both emotional and social support.

Inspired by Beardslee and colleagues (23) in the US, various programs have been developed in Australia, Canada, Finland, the Netherlands, and Norway in order to promote youth mental health and reduce risk factors linked to living with parents affected by a mental disorder (24), mostly affective disorders. Family Talk (25) was the first structured family-based preventive intervention, and because of its demonstrated efficacy, this program has been implemented in various countries worldwide (26). As an alternative to family approaches, other programs offer group-based cognitive–behavioral preventive interventions (27) and treatment coordination tailored for individual families (28). Web programs have also been tested. Programs vary in length and include very brief interventions made up of 1–3 sessions (26, 29). Some interventions focus only on parents instead of the whole family (30).

There is a great heterogeneity in the preventive programs for children as well as in their documented efficacy (17, 31). The common component across these preventive initiatives is the provision of psychosocial education on how to cope with parental mental disorder to families and children. However, further evaluation is required to examine what interventions work and for whom (e.g., sample characteristics), and through which mechanisms (e.g., program components and fidelity). Risk screening seems to be necessary to ascertain the type and intensity of support that best meets the risk profiles and needs of individual children and families (16).

At odds with the promising preventive initiatives in this field, many countries in Europe neglect these children’s needs or have no structured responses in terms of dedicated mental healthcare pathways or programs. The lack of prevention in many EU countries may stem from culture-mediated lack of consideration of the service users’ families (including siblings, partners, and children) in mental healthcare settings, and hence individual- or illness-centered care and treatment models. In addition, specific economic, organizational, and political issues may contribute to the lack of a child focus (i.e., resource distribution may be limited, poor or unavailable protocols for intervention, lack of collaboration between adult mental health services and the rest of the healthcare social care and lack of educational resources).

In 2015, Giovanni de Girolamo, M.D., initiated a joint application for funding from Horizon 2020 named PROCHILD. He created an international consortium with the aim to develop an international multi-site research project to evaluate and compare the effects of a very brief intervention across the nations collaborating in the consortium. The members of the consortium were experienced researchers and clinicians in the field of parental mental disorder and their children. The research and intervention model protocol described in this article is derived out of the PROCHILD protocol.

The aim of the present project is to provide a research and intervention model protocol to evaluate a very brief intervention for children of mentally ill parents adapted for a large-scale implementation. The intervention is based on an existing preventive strategy already adopted in the Netherlands and Norway called the “Child Talks” (29, 32, 33). The intention is to contribute to the current state of the art, by providing a model protocol for a feasible and widely replicable preventive intervention. This is feasible as it makes it possible to:

1. Implement a structured and brief preventive intervention targeted at families with children aged 6–18 years old and one or both parents suffering from mental disorders.
2. Evaluate the efficacy of a very brief intervention compared with treatment as usual.

## Method

### Participants

Participants should be diagnosed parents of minor children aged 6–18 years old where parents are receiving outpatient or inpatient treatment at mental health services for any mental disorders. A total of two to three treatment clinics in each participating country should serve as intervention sites. The minimum final sample size for each experimental condition should be N = 82.

### Recruitment

**Figure 1:**
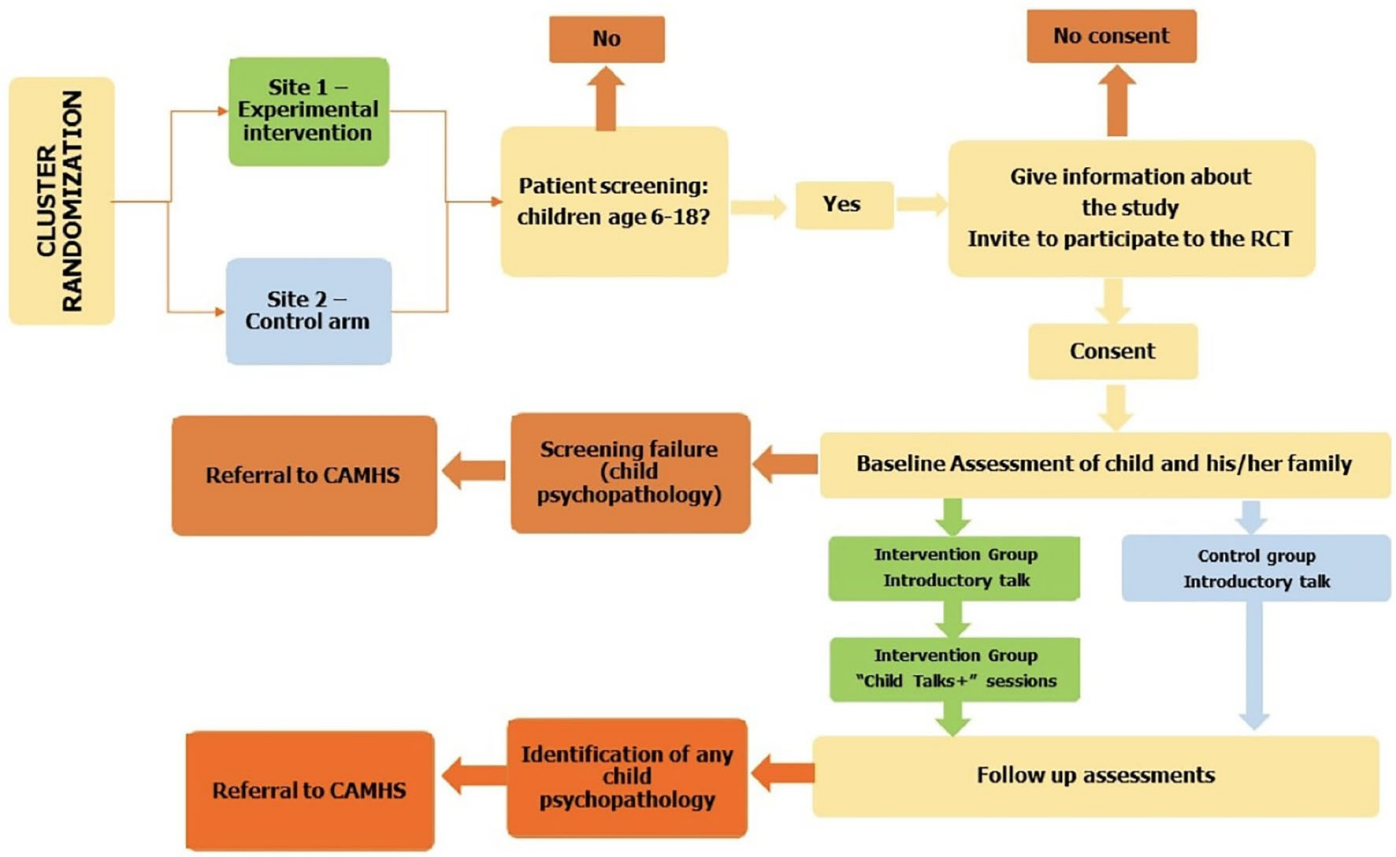
Study design flowchart.

The primary investigator of an international study should establish an international consortium with researchers from the COPMI field represented. Each of the participating researchers should have access to relevant clinical sites *via* university hospitals and clinics. In each country, there should be cluster randomization (site-randomization) of at least two to three participating adult services. Participants should be recruited through adult mental health services where parents are in treatment, inviting patients and their families to take part in the study as part of the treatment offered. Recruitment should be supported by all health care and social workers in each site, and posters and flyers should be available to inform parents. The intervention to be evaluated should be randomly assigned to the experimental or control condition (see **Figure 1** for details).

*Inclusion criteria:* Families should have at least one parent in treatment for any psychiatric disorder (according to DSM 5 or ICD-10 diagnostic criteria) and at least one child aged 6–18 years.

*Exclusion criteria:* Parents with current substance or alcohol addiction, who are acutely ill or actively suicidal parents, and parents with serious physical co-morbidities should be excluded, as these families need more intense interventions. Parents who are not able to provide consent/assent due to language or other difficulties and parents with an IQ ≤ 70 or indication of intellectual impairment should be excluded. Exclusion criteria regarding the child(ren) include ongoing psychotherapy and/or pharmacotherapy led by a Child and Adolescent Mental Health Service (CAMHS), as well as children in foster care or custody (even with grandparents).

### Procedure

A coordinator should explain the study in details to the potential participants and request their consent to contact the rest of the family. Subsequently, consent should be obtained from the participating family members. Participation by the whole family should be encouraged, although intervention can start even when only part of the family (at least one parent and one child) is willing to accept. In order for children (under the age of legal consent) to take part in the study, the parents’ consent is required.

Participants should be provided with a calendar-based sessions/assessment schedule and receive reminders for their appointments. For the intervention group, two facilitators (trained in the intervention strategies) should conduct each session. Trained personnel should conduct assessments for all groups.

### Design

**Figure 2:**
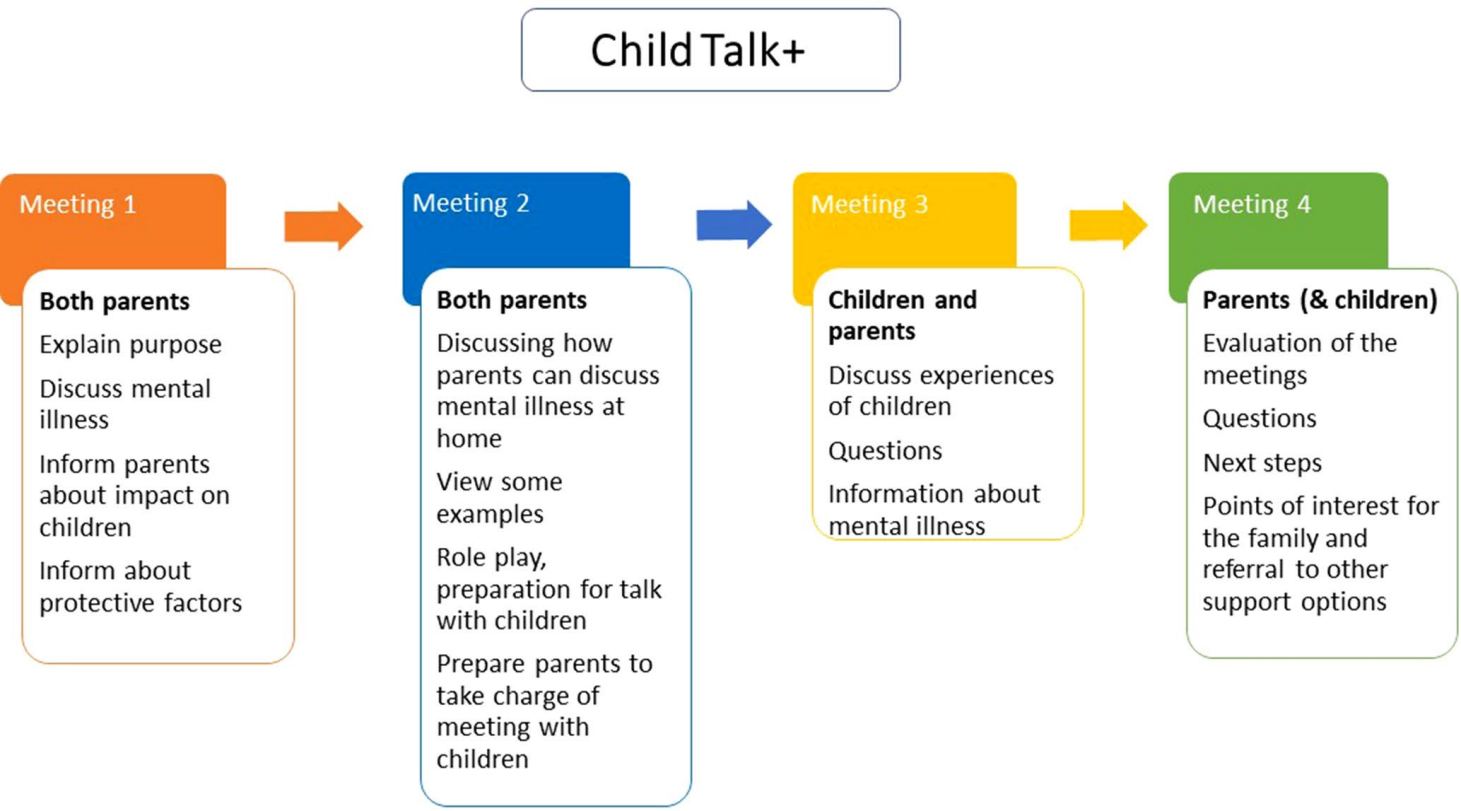
Intervention outline.

The design of the research is a cluster-randomized controlled trial evaluating the effects of an improved version of the Child Talk Intervention called Child Talk+. In each country, the participating adult services (clusters) should be randomized into to the following experimental conditions:

**Experimental group.** After the baseline assessment (T0), recruited patients/families in participating services should be given the manualized “CHILD TALK+” intervention (four weekly 45-minute sessions, with trained facilitators); see **Figure 2** for the outline. After the intervention, assessment should be repeated (T1). Parents and children should be followed up at 6 (T2), 12 (T3), and 18 (T4) months. The contents and language of sessions should be adapted to the type of parental disorder and the age group of the child(ren) (6–12 or 13–18 years).
**Control group.** After the baseline assessment (T0), participants should receive information session about the mental health of children living with a parent affected by mental disorder, including risk and resiliency factors for the child(ren). The rationale for this experimental condition is that several European countries have policies, which mandates health personnel to identify and provide care for children of parents with mental disorder. A true control condition with no information would force health personnel in such countries to break the laws, and therefore a control condition including information about the risks for the children is necessary to include such countries. After the introductory session, postintervention assessment should be conducted (T1). Participants should be followed up at 6 (T2), 12 (T3), and 18 (T4) months. Support material should be available, but with no active role of facilitators in transferring this information into family practice.
**Randomization.** Cluster randomization will buffer against contamination effects, and each cluster will only deliver intervention/no intervention representing one experimental condition. The trial should be conducted according to Good Clinical Practice, reported according to CONSORT guidelines, analyzed according to SPIRIT guidelines, and registered at www.clinicaltrials.gov.

### Intervention

Child Talks+ is an intervention where the mental health workers talk with the family about the situation of the children and their needs when a parent struggles with mental health issues. This intervention was developed in the Netherlands (32) and has been part of regular practice for two decades there. The updated intervention Child Talk+ comes with a manual that describes the process of carrying out four separate conversations/meetings; two initial conversations/meetings with the patient and possibly his/her partner, followed by two conversations/meetings with the patient (and partner) and the children involved (34).

**Meeting 1.** The initial meeting is preferably to be conducted with both parents/caregivers. In the beginning of the meeting, the purpose of conversation one should be explained to the parents/caregivers. The mental health workers should talk to the parents about the potential consequences of the mental disorder on their children and the family life. Parents should be given relevant information about the potential impact on the children, and they should be informed about possible protective factors.

**Meeting 2.** The second meeting is also conducted with both parents/caregivers. The purpose of this meeting is to supervise the parents and inform them about how they can discuss mental disorder at home. Examples on how to address these issues should be given, and the mental health workers should practice with the parents on how to talk to children about mental disorder, for instance, through role-play. At the end of meeting two, preparations for the next meeting should be done together with the parents/caregivers.

**Meeting 3.** In meeting three, the children also participate, together with the parents. The main purpose of this conversation is to get an overview of how the children are coping with the situation. The children’s experiences should be discussed. Another important purpose of this meeting is to give emotional support and to provide information about the parent’s mental disorder to the children (by the parents supported by the mental health worker) as prepared in meeting two. If the children have any questions, they should be answered by the parents or the mental health workers.

**Meeting 4.** The final meeting is with both parents/caregivers and the children if possible. The purpose of this meeting is to sum up and evaluate the previous meetings. Any questions the family may have should be addressed. The possible next steps for the family should also be discussed, and the mental health workers should assist the family in order to seek additional support if necessary. The intervention is illustrated in **Figure 2**.

The intervention allows the parents/patients to describe their children’s resources and vulnerability and to participate in planning how they want their child to be informed of the family situation. The intervention includes the children through questions about their understanding and experiences of the family situation, and the children’s view of what may improve their situation.

### Intervention Integrity

The professionals should follow the manual for the intervention and complete standard checklists (logbook) for each session to ensure this.

### Training

Training for facilitators should be provided according to manualized procedures. A set of booklets and slides should be created as consultation materials for both intervention and control conditions.

### Measures

Multiple informants, including the children themselves, will contribute to tap a variety of areas at baseline, postintervention, and follow-up assessments. All assessments will be conducted individually with each subject, and these include: child wellbeing, child resilience, feelings of guilt and shame, child problems and development status, parents’ evaluations of child developmental status, parent–child communication, child mental health literacy, and parenting competence. The following measures are suggested:

*Sociodemographic variables:* Parental gender, age, marital state, living situation, education, work, income, as well as parental diagnosis and severity of parental mental disorder. Diagnosis can be retrieved from clinical records. Diagnoses should be based on structured clinical interviews as SCID or MINI (35) and be codified with DMS IV, DSM 5 (36, 37), or ICD-10 (38). Severity of parental mental disorder parent should be retrieved from clinical records or GAF score (36). Children’s baseline characteristics should include age, gender, living with mentally ill parent or not, total number of siblings, and educational attainment.

*The health-related quality of life* (KIDSCREEN-27) (39). The KIDSCREEN-27 is a measure for health-related quality of life for children from 8–18 years of age. It contains 27 items building five subscales: physical wellbeing, psychological wellbeing, autonomy and parents, social support and peers, and school environment. A 5-point Likert response scale is used in all subscales. All scores are reported as T-values, with higher scores indicating higher health-related quality of life. KIDSCREEN-27 was found to be a reliable and valid measure of quality of life in children and adolescents (40). Answering the KIDSCREEN-27 requires 10–15 minutes.

*The Resilience Scale for Adolescence* (READ) (41). READ is a self-report questionnaire measuring resilience: the ability to handle stress and negative experiences. READ is a 28-item scale with positively formulated items organized in five subscales: personal competence, social competence, social support, family cohesion, and structured style. Statements are answered on a 5-point Likert scale from 1 (completely disagree) to 5 (completely agree). Higher scores indicate higher degrees of protective characteristics associated with resilience within each domain. Subscale scores are summarized into a total score for resilience. It takes 5 minutes to complete the questionnaire. READ shows adequate psychometric properties and promising validity when correlated with measures of mental difficulties (42).

*Guilt and Shame Questionnaire for Adolescents of Parents with Mental Illness* (GSQ-APMI; 3). This questionnaire includes 10 items, 5 items measuring shame, and 5 measuring guilt. Adolescents are to answer how often they have experienced feelings of guilt and shame, with answers on a 5-point Likert scale from ranging from 0 (never) to 5 (always). Reliability scores were found adequate in a previous study (43).

*The Strengths and Difficulties Questionnaire* (SDQ) (44). SDQ is a brief behavioral screening questionnaire for children aged 3–16 years old. The scale is composed of 25 items, divided between 5 scales: emotional symptoms, conduct problems, hyperactivity/inattention, peer relationship problems, and prosocial behavior. Statements are answered on a 3-point Likert scale ranging from not true, somewhat true, to certainly true. Reliability scores have been found adequate in previous studies (45).

*Parents’ Evaluations of Child Developmental Status* (PEDS) (46). PEDS is a 10-question measure. The first item is an open-ended question where parents describe any concerns they may have about their children in terms of behavior, learning, and development. In the following eight questions, the parents consider whether they have concerns in each developmental domain, and the final question probes any additional concerns. PEDS determines whether children are at a) high risk for developmental problems, b) moderate risk for developmental and/or mental health problems, c) limited risk but in need of in-office advice, or d) limited/no risk (47). Reliability scores have been found adequate in previous studies.

*Parent–Child Communication Scale* (48). The Parent–Child Communication Scale consists of one scale for children and one for parents. The child report consists of 10 items measuring children’s perceptions of their primary caregiver’s openness to communication. Statements are answered on a 5-point Likert scale from 1 (“almost never”) to 5 (“almost always”). The parent report reflects the child’s perception of the primary caregiver’s effort to maintain open communication with him/her. The child communication scale reflects the frequency with which the child communicates his/her feelings and problems with the primary caregiver.

*Children’s Mental Health Literacy Scale* (49). The scale examines children’s knowledge of mental disorder, recovery, and stigma. The scale consists of multiple choice questions developed for children of a parent with a mental disorder. The scale is currently being tested and will be ready for dissemination during the next year.

*Parenting Sense of Competence* (PSOC) (50). The PSOC is a 16-item measure intended to assess parents’ beliefs that they are capable of doing a good job parenting their child. It is comprised of two subscales and is rated on a 6-point scale from 1 (“strongly agree”) to 6 (“strongly disagree”). The efficacy subscale measures parents perceived competency (e.g., “being a parent is manageable, and my problems are easily solved”), while the satisfaction subscale measures parental satisfaction (e.g., “being a parent makes me tense and anxious”). Research on the PSOC has demonstrated adequate reliability and validity when used with parents of young children (50).

*User satisfaction.* The satisfaction of healthcare users should be evaluated by a scale tapping into issues related to challenges of being a parent with mental health problems.

### Sample Size and Statistical Power

Based on the results in Prchal et al. (51), the delta score, pre-post 4 months, of KIDSCREEN-27 in a single group (similar to an experimental group with four meetings) is equal to 4.28. Therefore, in a precautionary way, we hypothesized a lower difference pre-post of about 2.5 between the experimental group (four meetings) and the control group (one meeting). Considering the longitudinal design of this model study protocol, we hypothesized a preventive correlation between two evaluations (baseline-post treatment) of 0.5 so that the pooled SD of the scores changes will be 4.90. With such data and using a two-tailed paired t-test with confidence level of 95% and a power of 0.8, the estimated sample size is N = 60 for each group (experimental and control group). Considering a drop-out rate of 25%, the minimum sample size is of N = 160 (80 per group). Given that all children of the same sick parent can be involved in the trial, we also have to consider the ICC for the intra-family correlation. Although this ICC is likely to be lower, we hypothesized an ICC of 0.04. The average number of children in European families is quite variable, ranging from 1.2 in Portugal to 2.0 in France (2014 data). Since we also have to account for non-European countries, we hypothesize an average number of children per family of 1.5. The design effect in this case will be 1.02, increasing the sample size to about N = 164 (82 children per group). Finally, this sample size should be adjusted for the multicenter design of the study. DE is defined (52) as: DE = 1+(N/m−1)*ICC, where m is the number of sites, and ICC is the intra-class correlation coefficient (e.g., ICC = 0.03). Therefore, the minimum final sample size for each group will be equal to N = 82*DE.

### Statistical Analysis

Group comparisons on demographic variables can be carried out using ANOVA or chi-square tests, depending on whether the variables are continuous or categorical.

We suggest to test three specific questions about group differences over time: 1) Are there any group differences in change from pre- to post-interventions? 2) Are there any group differences in change from pre-intervention to follow-up? And 3) are there any group differences in change from post-intervention to follow-up? Rausch, Maxwell, and Kelley (53) argue that these specific questions should be analyzed using ANCOVA, controlling for the pre-score in all analyses to maximize power. We therefore suggest using ANCOVA and to use the pre-score as covariate in all analyses (53). In order for the ANCOVA to be valid, there should be no treatment group differences on pre-intervention measures. In order to test whether the intervention and control group were different at pre-intervention measures, we suggest using a one-way ANOVA. Effect sizes should be calculated according to suggested methods (54).

### Ethical Considerations

Each participating country should apply for ethical approval of the project from their relevant Regional ethics committees. There are several ethical dilemmas, which could be discussed in such application: a) what are the norms to which parents with mental disorders feel obliged to follow, when they try to be good parents; b) how far are they able to follow these norms, or when and why do they fail to follow them; and c) what are the cost of not intervening to stop the transgenerational transfer of mental disorder.

In cases of screening failure due to the child(ren)’s overt psychopathology or in the event that during the trial, or at follow-up assessment, serious concerns about the child(ren)’s psychopathology should emerge; the research team should act according to country-specific guidelines and norms, as agreed with regional ethical committees (i.e., referral to Child and Adolescent Mental Health Services).

### Dissemination

Data from projects using the present protocol should be shared and available for researchers *via* accepted data repositories. Principles for storing data in national databases and cross-national exchange should follow relevant laws and guidelines. Results should be published internationally and presented at conferences, as well as disseminated *via* international research groups and collaboratives.

## Results

Outcomes should be reported in terms of demographics, parent’s diagnosis, child behavioral and emotional problems, child wellbeing, family communication and functioning, as well as user satisfaction. The expectation would be to find greater and more sustained improvements for these outcomes in the intervention group than in the control condition, and test results should be presented.

## Discussion

Research projects based on the model study protocol presented in this paper will draw the attention of scientific and political communities toward children of mentally ill parents. This young segment of the population is presently almost completely neglected in most European national health policies, despite carrying the largest burden of disability in this age group. On a daily basis, this population faces social, biological, and environmental risk factors threatening their mental health.

The European context is not homogeneous, but only very few countries (e.g., Netherlands Norway, Sweden, Portugal, and Finland) are currently implementing protocols for children with parents affected by mental disorders. The rest of Europe implements at most, less structured initiatives in a few areas, with no data available in terms of evaluated clinical effectiveness (and costs). A universal approach has never been fully adopted. Thus, our model protocol is consistent and complementary as it aims to implement and test a very brief intervention for children of parents with mental disorders to prevent the onset of mental ill health, reduce disability and symptomatology, and ameliorate developmental disruption. Furthermore, other universal interventions may also be tested using this protocol.

The advance of the current state of the art is twofold. First, for those countries where the intervention has already been implemented, this study will i) offer the chance to test its efficacy through the replication of the results and ii) assess its external validity, exporting the model to other countries. Secondly, countries currently with no structured intervention will be able to implement it in their own health services, testing its efficacy, and evaluating its feasibility. The result of any national or international research projects based on the present model protocol could make a platform for establishing guidelines to be disseminated throughout Europe and beyond. Such guidelines would contribute to: a) recognize the needs of selective prevention initiatives, b) define the minimum requirements in formulating a protocol in each country, and c) ensure greater homogeneity of health policies in Europe and worldwide, regarding prevention and support for all minor children with parents suffering from severe mental disorders.

Improving youth wellbeing and good health is a key issue of current EU and WHO policies. The Child Talk+ intervention is primarily oriented toward promoting positive mental health for young people whose parents suffer from mental disorders. It aims at boosting existing service models. Interventions like this one will create an innovative, evidence-based intervention platform, which will provide a solid basis to improve existing healthcare systems. The present model protocol also opens the possibility to evaluate the sustainability, feasibility, and cost-effectiveness of a very brief intervention in different sites and countries. Results from such projects will inform policy makers and foster decisions that will increase the cost-effectiveness of care, thus improving the therapeutic management of patients with young children and help define preventive strategies for children’s wellbeing.

In particular, research projects based on the present model protocol will address the expected impact, as follows:

**Improved support and parenting among mentally ill parents**. By introducing evidence-based intervention strategies, improving early recognition of children’s ill health and malleable risk factors, this RCT will support a family approach in the current adult mental service. The intervention will decrease the potential impact of risk factors related to stressful environmental stimuli within the family, such as poor or absent communication of mental suffering, poor understanding of symptoms or disorders, insufficient parental care, and lack of clinical support or help-seeking. Patients will receive acknowledgement for their parenting role and support in their effort to take good care of their children although they have mental health problems. Its replicability will assist in creating a 21st century clinical framework including neglected children’s needs and to promote improved integration of healthcare.

**Improved wellbeing among COPMI** through supportive interventions that will help them cope with a demanding family environment (created by the fragility and complexity of a parent with mental disorder). This will enhance individual resources to cope with these stressors by providing information about parents’ mental disorder and giving families emotional and social support. It will also reduce negative experiences and negative emotions that could lead to feelings of intense discomfort. By limiting the negative effects of prolonged exposure to stress, the medium- and long-term impact will decrease the probability that youngsters’ psychological distress leads to mental disorder.

**Establish preventative strategies** favoring the mental dimension of healthy childhood by allowing youngsters to meet age-specific developmental goals. Research projects based on this model protocol will aid policy makers and service providers at local, national, and international levels concerning the policy, strategic, clinical, and organizational changes needed to ensure appropriate primary and secondary prevention programs. It will also help integrate treatment for this under-served clinical population, promoting the mental and physical dimensions of healthy childhood.

**Establish recommendations for the integrated treatment of patients** with mental disorders and their children. Research projects based on this model protocol will foster the development of recommendations for training programs targeted at clinicians and allied professionals on how to improve mental health care for patients who are also parents, as well as good collaboration between AMHS and CAMHS. Local variations in service structures, healthcare provision, and clinician training will be taken into account.

## Author Contributions

All authors have, CR, KD, GS, CL, TA, FS, AY, PC, RM, TS, MB, AS, GP and GG, contributed in the conceptualization of the model protocol with regard to the design and methods, as well as having read and commented on the manuscript text. CR, KD, GS, and CL have put the paper into writing. All authors have approved of the final version of the paper.

## Conflict of Interest Statement

The authors declare that the research was conducted in the absence of any commercial or financial relationships that could be construed as a potential conflict of interest.
